# The neural stem cell properties of Pkd2l1^+^ cerebrospinal fluid-contacting neurons *in vivo*

**DOI:** 10.3389/fncel.2022.992520

**Published:** 2022-09-09

**Authors:** Liang Cao, Ming-Zhi Huang, Qiang Zhang, Zhang-Rong Luo, Yi Zhang, Ping-Jiang An, Lei-Luo Yang, Wei Tan, Chun-Qing Wang, Xiao-Wei Dou, Qing Li

**Affiliations:** ^1^Department of Traumatic Orthopedics, The Affiliated Hospital of Guizhou Medical University, Guiyang, China; ^2^School of Clinical Medicine, Guizhou Medical University, Guiyang, China; ^3^Clinical Research Center, The Affiliated Hospital of Guizhou Medical University, Guiyang, China

**Keywords:** CSF-cNs, neural stem cell, spinal cord injury, Pkd2L1, cerebrospinal fluid-contacting neurons

## Abstract

The neural stem cells (NSCs) in the ventricular-subventricular zone of the adult mammalian spinal cord may be of great benefit for repairing spinal cord injuries. However, the sources of NSCs remain unclear. Previously, we have confirmed that cerebrospinal fluid-contacting neurons (CSF-cNs) have NSC potential *in vitro*. In this study, we verified the NSC properties of CSF-cNs *in vivo*. In mouse spinal cords, Pkd2l1^+^ CSF-cNs localized around the central canal express NSC markers. *In vitro*, Pkd2l1^+^ CSF-cNs form a neurosphere and express NSC markers. Activation and proliferation of CSF-cNs can be induced by injection of the neurotrophic factors basic fibroblast growth factor (bFGF) and vascular endothelial growth factor (VEGF) into the lateral ventricle. Spinal cord injury (SCI) also induces NSC activation and proliferation of CSF-cNs. Collectively, our results demonstrate that Pkd2l1^+^ CSF-cNs have NSC properties *in vivo* and may be involved in SCI recovery.

## Introduction

The usage of neural stem cell (NSC)-based therapy in spinal cord injury (SCI) offers promise for repair and recovery (Lu et al., 2012; Curtis et al., 2018; Rosenzweig et al., 2018). Indeed, SCI promotes a significant expansion of the NSC population as a result of increased neurosphere formation (Barnabe-Heider et al., 2010). However, the identity of the NSCs in the central canal of the spinal cord is disputable. A previous study uncovered that NSCs are located in ependymal cells and, thus, serve as cell transplantation after SCI (Llorens-Bobadilla et al., 2020). However, another failed to confirm that ependymal cells have neural stem-cell functions (Shah et al., 2018a). Therefore, identification of the NSCs in the central canal of the spinal cord is vital for NSC-based therapy in SCI.

Cerebrospinal fluid-contacting neurons (CSF-cNs) are a subset of neurons that directly contact cerebrospinal fluid (CSF) and traverse the parenchyma (Vigh and Vigh-Teichmann, 1973). Although these cells were discovered nearly 100 years ago, their functions are not well understood. Based on their location, CSF-cNs were once considered a sensory “organ” for detecting CSF composition and flow. Indeed, in recent studies, CSF-cNs have been found to exhibit an intuitive mechanosensory function for detecting CSF flow through Polycystic kidney disease 2-like-1 (Pkd2l1) (Sternberg et al., 2018). Conversely, CSF-cNs have been shown to respond to mechanical stimulation and pH reduction through ASIC3 channels (Jalalvand et al., 2016). In addition, there is evidence that CSF-cNs play an essential role in detecting spinal curvature (Lu et al., 2020; Wu et al., 2021).

As early as 2003, a study reported that a highly plastic population of GABAergic CSF-cNs expressed GAD and polysialylated neural cell adhesion molecule (PSA-NCAM) in the rat spinal cord, and PSA-NCAM is a marker of cellular plasticity (Stoeckel et al., 2003). Other researchers also discovered many newly formed neurons clustered around the central canal after lamprey spinal cord transection, and this phenomenon still existed even 7 weeks after spinal cord transection; in the study, it was suggested that these newborn neurons may be CSF-cNs (Zhang et al., 2014). Furthermore, a similar phenomenon has also been reported in eels (Dervan and Roberts, 2003; Rodicio et al., 2008). In addition, Pkd2l1^+^ CSF-cNs also express the transcription factor Nkx6.1, a homeodomain-containing protein that differentiates ventral progenitors into somatic motor neurons and interneurons (Djenoune et al., 2014), and Djenoune et al. (2017) also found that Gata2^+^ CSF-cNs exhibited molecular and physiological characteristics of immature neurons in newborn and young rats. Moreover, another study identified that CSF-cNs expressed Doublecortin (Dcx) and Nkx6.1 (two markers of immature neurons), as well as neuron-specific nuclear protein (NeuN), with this phenotype persisting in 12-month-old animals (Orts-Del’Immagine et al., 2014). Therefore, this work illustrates that these CSF-cNs maintain a persistent low-differentiated state, although the continuous maturation of CSF-cNs happens after birth (Orts-Del’Immagine et al., 2017). A recent study revealed that the depletion of CSF-cNs reduced the number of endogenous neural progenitor cells and impaired their motor function in spinal cord-injured rats; this suggests that these CSF-cNs may play a crucial role in functional recovery following SCI (He et al., 2021). In our previous study, we prospectively isolated spinal cord CSF-cNs *in vitro* and confirmed that they had the properties of NSCs; Indeed these neurospheres could differentiate into neurons, astrocytes, and oligodendrocytes in the *in vitro* culture (Wang et al., 2021).

The current study aimed to further explore whether these Pkd2l1^+^ cells maintain their stem cell properties *in vivo* using several interventions with CSF-cNs, including SCI, growth factors, and NSC culture conditions. The findings of this study provide fundamental insights into the use of CSF-cNs as endogenous NSCs for the repair of SCI.

## Experimental procedures

### Animal model

C57BL/6 mice aged 6–8 weeks were obtained from the Experimental Animal Center of Guizhou Medical University (license no. SCXK [Qian] 2018-0001). All animal experiments were approved by the Animal Care and Use Committee of Guizhou Medical University.

### Spinal cord injury model

All mice were anesthetized using 1.25% Avertin. The clip compression model of SCI was conducted as previously described (Cheriyan et al., 2014). Briefly, this model of SCI involves a laminectomy at the tenth thoracic vertebra of the spine. The clip is closed around the spinal cord at the tenth thoracic *vertebra* level, which then compresses the cord for 15 s. In the sham group, the cord was not compressed by the clip. Each group was done in triplicate at least.

### Stereotactic injection of vascular endothelial growth factor + basic fibroblast growth factor

Injection of vascular endothelial growth factor (VEGF) + basic fibroblast growth factor (bFGF) into the right ventricle was predominantly carried out as previously described (Shah et al., 2018a). The animals were anesthetized by 1.25% Avertin and, subsequently, mounted into a stereotaxic frame. The right lateral ventricle was injected with 3 ul PBS as a control or VEGF (1 mg/mL; Thermo Fisher Scientific, Waltham, MA, United States) and bFGF (1 mg/mL; Bio-Techne/R&D systems, United States) dissolved in PBS for growth factor stimulation. The needle was kept in place for 20 min following the injection. The spinal cord tissues were collected 1 week following VEGF/bFGF treatment. Each group was done in triplicate at least.

### Immunofluorescence staining

The animals were euthanized by Carbon Dioxide Euthanasia. The spinal cord tissue was fixed with 4% PFA at 4°C overnight, and the tissues were subsequently dehydrated with 30% sucrose gradients and embedded in OCT. The samples were penetrated with 0.25% Triton X-100 for 10 min and blocked with 1% BSA for 1 h. The sections were incubated with primary antibody at 4°C overnight. The primary antibodies included Nestin (sc23927,1:200, Santa Cruz Biotechnology, Dallas, TX, United States), Pkd2l1 (AB9084, 1;700, Merck Millipore, MA, United States), Sox2 (1:400, CST, Danvers, MA, United States), Dcx (1:200, Santa Cruz Biotechnology, Dallas, TX, United States), Gfap (1:300, CST, Danvers, MA, United States), Ki-67 (1:1000, Abcam, Cambridge, United Kingdom). After rinsing three times with PBS, the sections were incubated with secondary antibodies (goat anti-rabbit Alexa Fluor 488,1:500, CST, Danvers, MA, United States or goat anti-mouse Alexa Fluor 594, 1:500, CST, Danvers, MA, United States) in the dark for 1 h and then counterstained with DAPI.

### Cell culture

The procedure for the isolation of CSF-cNs has been described in our previous studies (Wang et al., 2021). In brief, lentiviral particles harboring pLVX-UbC-rtTANgn 2:2 A: PKD2L1 (titers ranged 1_106 to 1_107 colony-forming unit, 10_L) were prepared and applied to CSF-cNs. Between 72 and 74 h after the application of lentiviral particles, the CSF-cNs were treated twice with puromycin (0.5–1 g/mL, Gibco, Thermo Fisher Scientific, Waltham, MA, United States). We selected CSF-cNs with green fluorescence as NSCs. Single-cell suspension was maintained in 24 well ultra-low adhesion culture plates at a volume of 400 ul fresh serum-free neural culture solution per well at a temperature of 37°C in a 5% CO_2_ incubator. Every 3 days, 200 ul of the serum-free medium was added to each well until the cell reached 80% confluent, the cells were passaged. The neurospheres formed by the CSF-cNs of passage 3 were seeded in climbing slices coated with PDL and grown naturally for 12 h. Cell adhesion was observed and recorded. For cell differentiation, CSF-cNs were cultured in fresh neural culture solution with 2% fetal bovine serum (FBS) (Gibco, Thermo Fisher Scientific, Waltham, MA, United States), 1% GlutaMAX (Gibco, Thermo Fisher Scientific, Waltham, MA, United States), and 1% penicillin-streptomycin (Gibco, Thermo Fisher Scientific, Waltham, MA, United States) for 7 days.

As to the NSCs of ventricular-subventricular zone, we micro-dissected the lateral wall of lateral ventricles from C57BL/6 mice (within 24 h of birth). Then the tissue was enzymatically dissociated with papain at 37°C for 18 min. We obtained the single cell suspension through a 70 mm Nylon cell strainer. Single-cell suspension was maintained in 24 well ultra-low adhesion culture plates at a volume of 400 ul fresh serum-free NSC culture solution per well at a temperature of 37°C in a 5% CO_2_ incubator. Every 3 days, half of the serum-free medium was added to each well until the cell reached 80% confluent, the cells were passaged.

NE-4C (CL-0660, Procell, Wuhan, China) were maintained in minimum essential medium (MEM) with 10% FBS (Gibco, Thermo Fisher Scientific, Waltham, MA, United States), 1% penicillin-streptomycin (Gibco, Thermo Fisher Scientific, Waltham, MA, United States) at 37°C in a 5% CO_2_ incubator. For cell passage, we used Accutase (Innovative cell technologies, CA, United States) to detach them every 4 days and transferred into new poly-D-lysine (PDL)-coated dishes.

### Cell immunofluorescence

The supernatant was discarded, and the CSF-cNs were then washed in PBS three times. Following this, the cells were fixed with 4% PFA for 15 min at room temperature. The cells were washed in PBS three times again before blocking (10% goat serum and 0.3% TritonX-100 in PBS) for 1 h. All antibodies were diluted using blocking buffer. For double immunofluorescence, two primary antibodies were used sequentially, and the cells were incubated overnight with primary antibodies at 4°C; the following day, the cells were warmed for 30 min at room temperature and washed three times for 5 min in PBS. The cells were incubated for 2 h with secondary antibodies After three further washes in PBS, the cell nuclei were stained with DAPI. Finally, we observed the results using a fluorescence microscope.

### Western blot

Similarly, three different cell types (CSF-cNs, SVZ-NSCs, and NE-4C) were washed with cold PBS for two times and centrifuged at 1,000 rpm for 5 min to obtain cell pellet. Then these cells were lysed in RIPA lysis buffer (RIPA, Beyotime, Shanghai, China). The protein concentration was measured with BCA assays (Thermo Fisher Scientific, Waltham, MA, United States). The proteins were separated by SDS-polyacrylamide gel electrophoresis and transferred to a polyvinylidene difluoride (PVDF) membrane. We used 5% milk to block the membrane at room temperature for 90 min. PVDF membrane and the primary antibody were incubated overnight at 4°C. After washing with *Tris*-buffer saline tween (TBST) with three times, then the PVDF membrane was incubated with horseradish peroxidase (HRP) conjugated anti-mouse IgG and anti-rabbit IgG (1: 5000, Proteintech, Wuhan, China) for 1 h. Subsequently, we visualized these immunolabeled bands with an enhanced chemiluminescence reagent (Thermo Fisher Scientific, Waltham, MA, United States).

The primary antibodies included Nestin (66259-1-Ig,1:500, Proteintech, Wuhan, China), Pkd2l1 (13117-2-AP, 1: 500, Proteintech, Wuhan, China), Sox2 (66411-1-Ig,1: 1000, Proteintech, Wuhan, China), β-Actin (20536-1-AP, 1-10000, Proteintech, Wuhan, China).

### Quantitative real-time PCR

In this study, three different cells (CSF-cNs, SVZ-NSCs, and NE-4C) and spinal cord of mice were used for qPCR. CSF-cNs, NE-4C, and SVZ-NSCs were washed with PBS and centrifuged (1,000 rpm, 5 min) to obtain cell precipitation. Briefly, the total RNA was extracted using MolPure^®^ Cell/Tissue Total RNA Kit (Yeasen Biotechnology, Shanghai, China). Then, we polyadenylated and reverse-transcribed the RNA into complementary DNA using a poly(T) adapter following manufacturer instructions. Real-time PCR was done using a thermal cycler under the following parameters: a 5-min initial denaturation step at 95°C; 44 cycles at 95°C for 15 s; 55°C for 30 s; 72°C for 20 s. We subjected each sample to the entire experimental procedure in triplicate. Table 1 lists the primers specific for mRNA.

**TABLE 1:** Primer sequences.

Gene	Primer	Sequence (5′-3′)
*Pkd2l1*	Forward	TACCTCAGCAGCGTCTGGAACA
	Reverse	CTGCATACGTGTCTGGCTGTTG
*Nestin*	Forward	AGGAGAAGCAGGGTCTACAGAG
	Reverse	AGTTCTCAGCCTCCAGCAGAGT
*Gfap*	Forward	CACCTACAGGAAATTGCTGGAGG
	Reverse	CCACGATGTTCCTCTTGAGGTG
*Ki67*	Forward	GAGGAGAAACGCCAACCAAGAG
	Reverse	TTTGTCCTCGGTGGCGTTATCC
*Dcx*	Forward	CTGACTCAGGTAACGACCAAGAC
	Reverse	TTCCAGGGCTTGTGGGTGTAGA
*Sox2*	Forward	AACGGCAGCTACAGCATGATGC
	Reverse	CGAGCTGGTCATGGAGTTGTAC
*Gapdh*	Forward	CATCACTGCCACCCAGAAGACTG
	Reverse	ATGCCAGTGAGCTTCCCGTTCAG

### 5-Ethnyl-2′-deoxyuridine staining and imaging

To label proliferating cells following SCI or growth factor infusions, 5-ethnyl-2′-deoxyuridine (EdU) (Riobio, Guangzhou, China) was injected intraperitoneally (50 mg/kg). The tissue processing is similar to Immunofluorescence section. After Immunofluorescence staining, these sections were stained for EdU using cell-light EdU Apollo567 kit (Riobio, Guangzhou, China) according to the manufacturer’s instructions. We observed the results using a Leica confocal microscope.

### Nissl staining and stereological analysis

The spinal cord sections were washed with distilled water and incubated in Nissl staining solution (Solarbio, Beijing, China) for 1 h at 37°C. Then sections were incubated Nissl Differentiation for a few seconds and dehydrated with 100% ethanol twice for 2 min. Unbiased stereology was used to estimate the number of Nissl body numbers in the spinal cord using the optical dissector method. Furthermore, the region of gray matter surrounding the central canal (lamina X) was defined as the region of interest (ROI). The counting frame was 50 × 50 um, and the sampling grid size was 100 × 100 um.

### Statistical analysis

All experimental results were conducted in triplicate. ImageJ pro was used to count the cell numbers. For comparisons of groups across multiple groups, a one-way analysis of variance (ANOVA) was used. Mann–Whitney test was used for comparisons of groups across two groups. Data were represented and analyzed in Prism 8.4. A level of *p* < 0.05 was considered statistically significant.

## Results

### Cerebrospinal fluid-contacting neurons of the central canal of the spinal cord expressed neural stem cell markers *in vivo*

**FIGURE 1:**
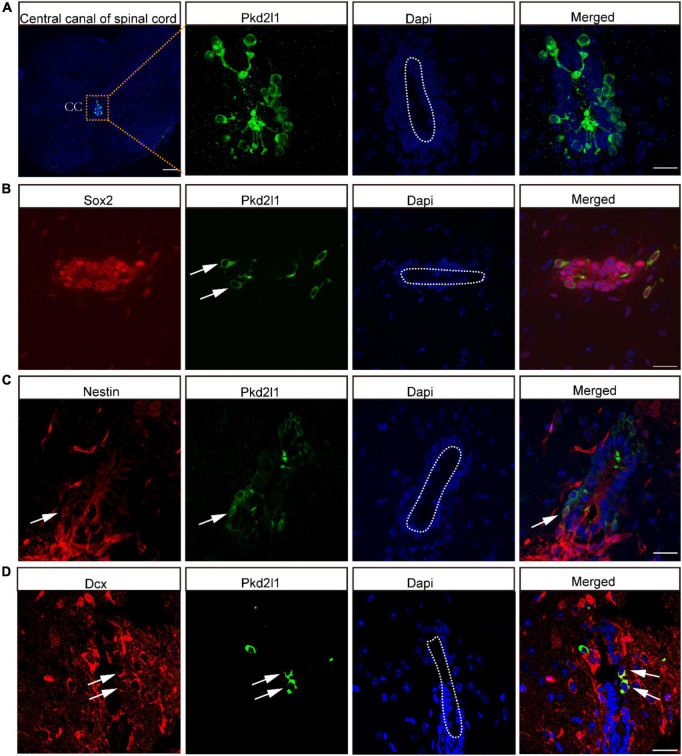
Cerebrospinal fluid-contacting neurons (CSF-cNs) expressed neural stem cell (NSC) markers *in vivo*. **(A)** Cross-section of the spinal cord and CSF-cNs of the central canal. **(B)** Representative images showed that CSF-cNs expressed NSC markers for Sox2. **(C)** Representative images showed that CSF-cNs expressed NSC markers for Nestin. **(D)** Representative images displayed that CSF-cNs expressed neuronal migration markers for Dcx. White arrows: Nestin, Sox2, and Dcx positive CSF-cNs. Scale bar represents 100 μm.

There is a cluster of CSF-cNs located in the subependymal layer of the central canal. These cells were found to express the specific marker Pkd2l1 (Figure 1A), even though CSF-cNs had been discovered approximately a century ago. Certain physiological functions of CSF-cNs are gradually being revealed owning to the identification of Pkd2l1 in 2006. In our previous study, CSF-cNs were confirmed to have the properties of NSCs *in vitro*. In this study, we performed these studies in adult mouse spinal cord to explore whether this unique characteristic of CSF-cNs also existed *in vivo*. We observed that these Pkd2l1^+^ cells expressed NSC markers such as Sox2 and Nestin (Figures 1B,C).

Interestingly, these co-labeled cells were located away from the central canal of the spinal cord. In addition, we also found that some Pkd2l1^+^ cells were co-stained for Dcx, which is a neuronal migration marker (Figure 1D) and is considered by some researchers a well-established marker for immature neurons.

### Cerebrospinal fluid-contacting neurons can proliferate and differentiate *in vitro* under pro-growth conditions

**FIGURE 2:**
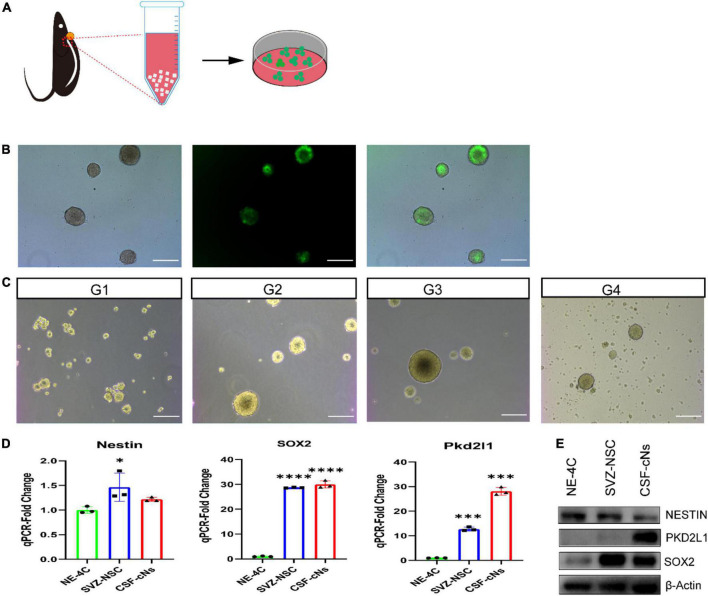
Cerebrospinal fluid-contacting neurons (CSF-cNs) formed neurospheres and expressed neural stem cell markers. **(A)** Illustration of CSF-cNs isolation and screening with the addition of lentiviral particles. **(B)** Target CSF-cNs displayed green fluorescent. **(C)** CSF-cNs were able to form neurospheres and passage at least four generations. **(D,E)** qRT-PCR and western blot demonstrated that CSF-cNs have the similar stem cell genes expression compared to NE-4C, SVZ-NSCs, but these cells strongly expressed PKD2L1. *p < 0.05, ^***^p < 0.001, ^*⁣*⁣**^p < 0.0001. Scale bar represents 100 μm in panels **(B–C)**.

**FIGURE 3:**
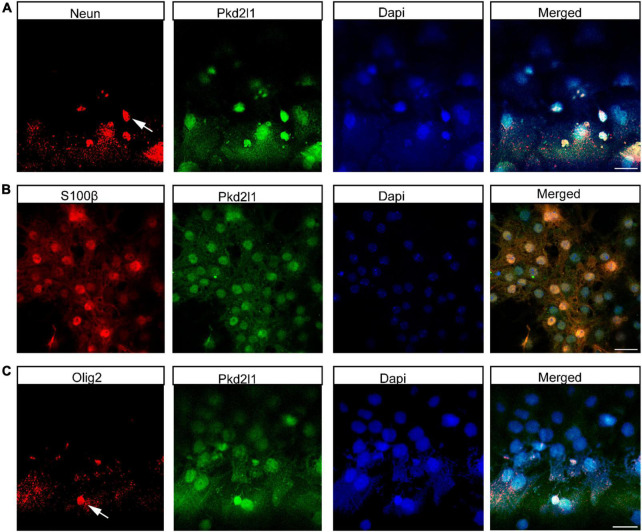
Cerebrospinal fluid-contacting neurons (CSF-cNs) have tripotential differentiation capacity *in vitro*. **(A)** Immunofluorescence analysis showed that CSF-cNs differentiated into NeuN-expressing neurons. **(B)** Immunofluorescence analysis displayed that CSF-cNs differentiated into S100β-expressing astrocytes. **(C)** Immunofluorescence analysis showed that CSF-cNs differentiated into Olig2-expressing oligodendrocytes. Scale bar represents 50 μm in panels **(A–C)**.

Generally, NSCs can form clonal colonies under growth factor conditions. To test the colony-forming capacities of CSF-cNs *in vitro*, we collected CSF-cNs from the microdissected cervical spinal cord and plated them under NSC culturing conditions. We further purified them using the screening of lentiviral particles harboring pLVX-UbC-rtTANgn2:2A: Pkd2l1 (Figure 2A). In this case, these target CSF-cNs showed green fluorescence under a fluorescent microscope (Figure 2B). Furthermore, within 1 week, we observed robust neurosphere formation. Moreover, these neurospheres can be expanded and passaged at least four generations (Figure 2C). In addition, to test whether these CSF-cNs have the unique characteristic compared to these widely accepted NSCs [NE-4C, ventricular subventricular NSCs (V-SVZ-NSCs)]. We validated their difference though western blot and quantitative real-time PCR (qRT-PCR). These three types of cells had the similar expression pattern of NSC genes (Sox2, Nestin). CSF-cNs had the high expression of Pkd2l1, while others did not (Figures 2D,E). To examine the multipotent capability of CSF-cNs, we induced cell differentiation by retracting EGF and FGF-2 from the medium. After 7 days, these dissociated CSF-cNs could differentiate into NeuN-expressing neurons (Figure 3A), S100β-expressing astrocytes (Figure 3B), and Olig2-expressing oligodendrocytes (Figure 3C).

### Growth factor injection results in cerebrospinal fluid-contacting neurons activation

**FIGURE 4:**
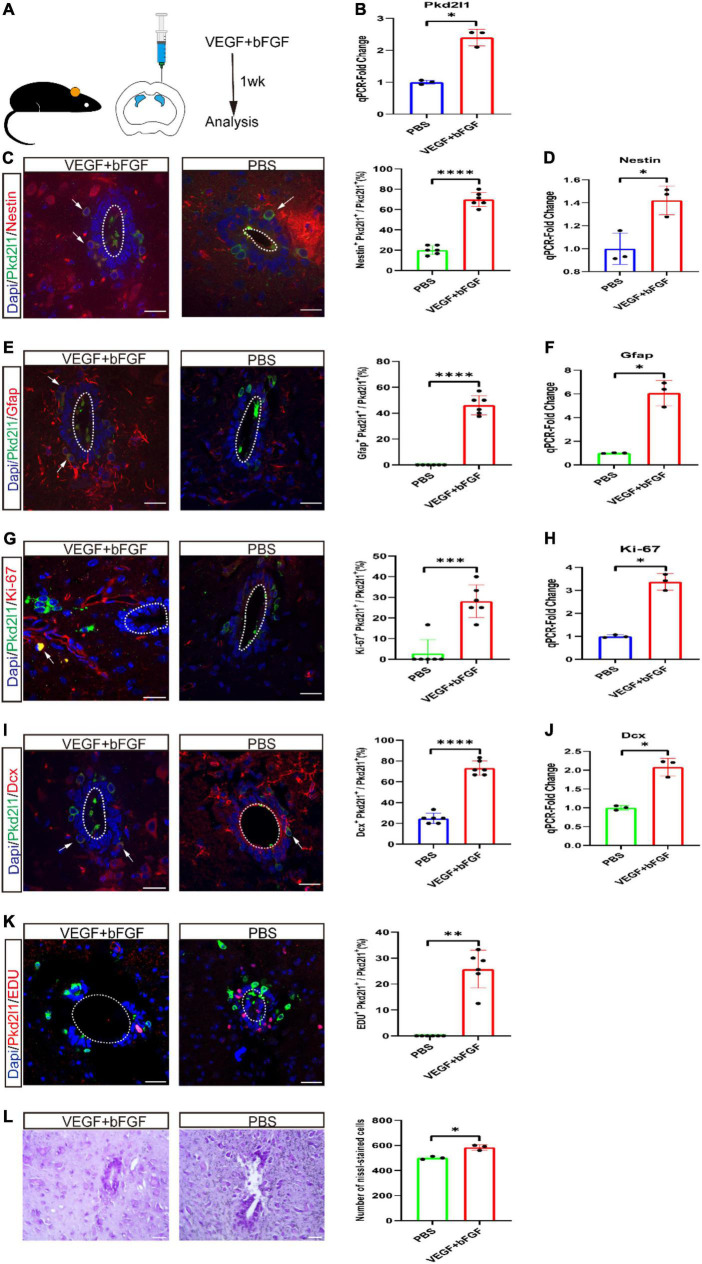
Cerebrospinal fluid-contacting neurons (CSF-cNs) activating proliferative or neurogenic potential after ventricular vascular endothelial growth factor (VEGF) and basic fibroblast growth factor (bFGF) injection. **(A)** VEGF and bFGF or PBS were injected into the right lateral ventricle by stereotaxic apparatus. **(B)** qRT-PCR demonstrated an obvious increase in PKD2L1 expression following infusion of VEGF and bFGF compared to PBS (*p < 0.05, *n* = 3). **(C,D)** Representative images displayed a robust increase in Nestin- (red) and Pkd2l1 (green) marked cells in the central canal following infusion of VEGF and bFGF compared to PBS. qRT-PCR demonstrated an apparent increase in Nestin expression following infusion of VEGF and bFGF compared to PBS. **(E,F)** Pkd2l1 (green) positive cells in the central canal expressed GFAP (red) following infusion of VEGF and bFGF compared to PBS. qRT-PCR demonstrated an obvious rise in Gfap expression following infusion of VEGF and bFGF compared to PBS. **(G,H)** The proportion of Ki-67 (red) and Pkd2l1 (green) marked cells in the central canal significantly increased following infusion of VEGF and bFGF compared to PBS. qRT-PCR demonstrated a significant increase in Ki-67 expression following infusion of VEGF and bFGF compared to PBS. **(I,J)** Representative images displayed a significant increase in Dcx (red) and Pkd2l1 (green) marked cells in the central canal following infusion of VEGF and bFGF compared to PBS. qRT-PCR demonstrated an obvious increase in Dcx expression following infusion of VEGF and bFGF compared to PBS. **(K)** Representative images demonstrated that Pkd2l1^+^ cells (green) incorporated EdU (red) at 1 week following infusion of VEGF and bFGF compared to PBS. **(L)** Representative images indicated a significant increase in Nissl-positive cells at 1 week following infusion of VEGF and bFGF compared to PBS. White arrows: double-labeled cells. *p < 0.05, ^**^p < 0.01, ^***^p < 0.001, ^*⁣*⁣**^p < 0.0001. The scale bar represents 20 μm **(C,E,G,I,K,L)**.

To explore whether these Pkd2l1^+^ cells could be activated under growth factor conditions *in vivo*, either VEGF + bFGF or PBS were syringed into the right lateral ventricle in equal volumes (Figure 4A). After 1 week, we observed there was an obvious increase in Pkd2l1 expression following infusion of VEGF and bFGF compared to PBS (*p* < 0.05, *n* = 3, Figure 4B). We also checked the cellular reactivity of Pkd2l1^+^ cells. Unsurprisingly, we observed a relatively significant increase in Pkd2l1^+^ Nestin^+^ co-expressed cells following the VEGF + bFGF infusion compared with the PBS group (*p* < 0.0001, *n* = 6, Figure 4C). qRT-PCR analysis displayed that a significant increase in Nestin expression following infusion of VEGF and bFGF (*p* < 0.05, *n* = 3, Figure 4D). Additionally, the Pkd2l1^+^ Gfap^+^ co-labeled cells also noticeably increased after VEGF + bFGF administration (*p* < 0.0001, PBS vs. VEGF + bFGF, *n* = 6, Figure 4E). However, as reported in previous literature, CSF-cNs do not express Gfap (Petracca et al., 2016). Correspondingly, qRT-PCR analysis represented that a significant rise in Gfap expression following infusion of VEGF and bFGF (*p* < 0.05, *n* = 3, Figure 4F). Notably, VEGF + bFGF treatment elicited not only relatively higher percentages of proliferative Pkd2l1^+^ Ki-67^+^ co-expressed cells but also of Pkd2l1 Dcx^+^ immature neurons (*p* < 0.001, PBS vs. VEGF + bFGF, *n* = 6, Figures 4G,I). Moreover, Pkd2l1^+^ EdU^+^ co-expressed cells also confirmed proliferation, and Nissl staining showed a visible increase in cell number (Figures 4K,L). qRT-PCR analysis showed a significant difference in Ki-67 and Dcx expression following infusion of VEGF and bFGF. At the end, we analyzed these data and qualified these changes (*p* < 0.05, *n* = 3, Figures 4H,J).

### Spinal cord injury induces the proliferation of cerebrospinal fluid-contacting neurons

**FIGURE 5:**
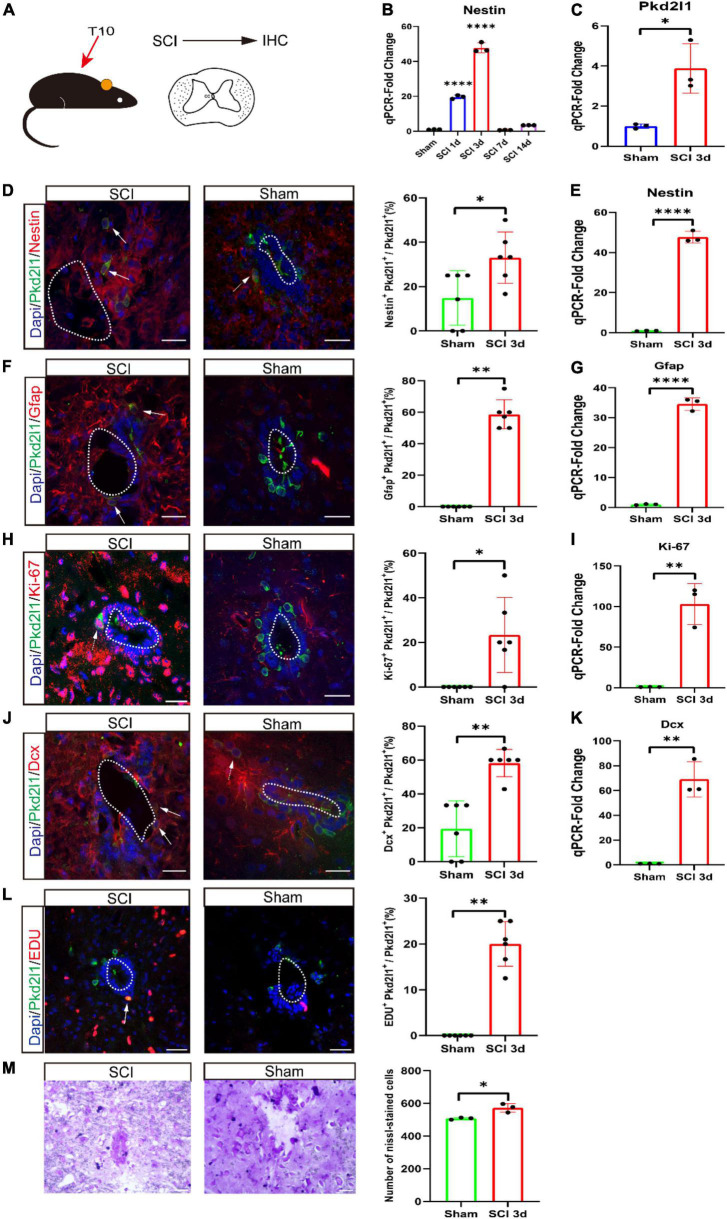
Cerebrospinal fluid-contacting neurons (CSF-cNs) activating proliferative or neurogenic potential after spinal cord injury (SCI). **(A)** SCI was performed at the T10 level, and IHC was analyzed. IHC, immunohistochemistry. **(B)** qRT-PCR analysis of Nestin expression in the spinal cords at different stages after SCI. **(C)** qRT-PCR demonstrated a significant increase in Pkd2l1 expression at 3 days after SCI compared to Sham. **(D,E)** Upregulation of Nestin (red) in Pkd2l1 positive cells (green) at 3 days post-SCI. Quantification of Nestin co-expression in PKD2L1 positive cells at 3 days post-SCI compared to sham. qRT-PCR demonstrated a significant rise in Nestin expression at 3 days after SCI compared to Sham. **(F,G)** The increase in expression of Pkd2l1 positive cells (green) at 3 days post-SCI. Quantification of Gfap co-expression in Pkd2l1 positive cells at 3 days post-SCI compared to sham. qRT-PCR demonstrated a significant increase in Gfap expression at 3 days after SCI compared to Sham. **(H,I)** Representative images demonstrating that Pkd2l1^+^ cells (green) start to proliferate 3 days post-SCI. Quantification of Ki-67 (red) co-expression in Pkd2l1 positive cells at 3 days post-SCI compared to sham. qRT-PCR demonstrated a significant raise in Ki-67 expression at 3 days after SCI compared to Sham. **(J,K)** Pkd2l1 positive cells (green) of the central canal were marked by robust Dcx^+^ (red) at 3 days post-SCI. Quantification of Dcx co-expression in PKD2L1 positive cells at 3 days post-SCI compared to sham. qRT-PCR demonstrated an obvious increase in Dcx expression at 3 days after SCI compared to Sham. **(L)** Representative images demonstrated that Pkd2l1^+^ cells (green) start to proliferate 3 days post-SCI. **(M)** Representative images demonstrated the increase in the number of Nissl-stained cells at 3 days after SCI compared to Sham. White arrows: double-labeled cells. *p < 0.05, ^**^p < 0.01, ^*⁣*⁣**^p < 0.0001. Scale bar represents 20 μm **(D,F,H,J,L,M)**.

There is a possibility that SCI can induce cell proliferation and cause NSCs at the lesion site to enter neurogenic reprogramming again. Some dormant NSCs will be activated after SCI. First, we performed the SCI mouse model at the T10 level (Figure 5A). Next, we validated the Nestin expression at different time point (1, 3, 7, and 14 days) following SCI using qRT-PCR. We observed that the Nestin expression reached its peak at 3 days after SCI (*p* < 0.05, *n* = 3, Figure 5B). Consequently, we also verified the expression of Pkd2l1 at 3 days of post-injury, qRT-PCR result displayed there was an obvious increase in Pkd2l1 expression (*p* < 0.05, *n* = 3, Figure 5C). To test whether these Pkd2l1^+^ cells could be activated by an SCI, we examined the molecular response of Pkd2l1^+^ cells to the NSC markers Nestin and Gfap at 3 days post-injury (DPI). Predictably, we observed that the percentage of Pkd2l1^+^ Nestin^+^ co-expressed cells significantly increased following SCI treatment (*p* < 0.05, *n* = 6, Figure 5D). qRT-PCR analysis displayed a significant rise in Nestin expression following SCI (*p* < 0.0001, *n* = 3, Figure 5E).

Similarly, the density of Pkd2l1^+^ Gfap^+^ co-expressed cells in the SCI group clearly exceeded the density in the sham group, for which few co-labeled cells could be detected (*p* < 0.01, *n* = 6, Figure 5F). qRT-PCR result showed a substantial increase in Gfap expression following SCI (*p* < 0.0001, *n* = 3, Figure 5G). In the SCI group, we also found that the percentage of Pkd2l1^+^ Ki-67^+^ and Pkd2l1^+^ Dcx^+^ co-stained cells were visibly higher than in the sham group (*p* < 0.05, *n* = 6, Figures 5H; *p* < 0.01, *n* = 6, Figure 5J). EdU incorporation assay indicated that Pkd2l1 positive cells proliferated after SCI, and Nissl staining also demonstrated an increase in cell number (Figures 5L,M). qRT-PCR analysis exhibited a significant augmentation in Ki-67 and Dcx expression following SCI (*p* < 0.01, *n* = 3, Figures 5I,K). This data indicates that SCI enhanced the proliferation of CSF-cNs and activated the potential of NSCs in the adult spinal cord.

## Discussion

A previous study demonstrated that the spinal cord of the adult mammalian central nervous system contains multipotent stem cells (Weiss et al., 1996). However, the sources of NSCs in the spinal cord remain unclear. Although the mammalian spinal cord’s central canal is widely accepted as an NSC niche (Dromard et al., 2008; Navarro Negredo et al., 2020), the identity of these NSCs is disputable. Previous research suggested that ependymal cells may be candidates for these NSCs (Ortiz-Álvarez et al., 2019; Redmond et al., 2019), but, conversely, other recent research has indicated that ependymal cells have a low probability of functioning as NSCs (Shah et al., 2018b; Xue et al., 2021). In comparison, CSF-cNs are located in the subependymal layer of the spinal cord’s central canal, their dendrites protrude into the central canal lumen, and their axons traverse the parenchyma. According to prior studies, these cells exhibit immature characteristics during development (Djenoune et al., 2014; Orts-Del’Immagine et al., 2017) and, in our previous studies, we first reported an *in vitro* culture of CSF-cNs and identified them as candidates for spinal cord NSCs (He et al., 2021; Wang et al., 2021). To further confirm the biological function of CSF-cNs, this study examined CSF-cNs *in vivo*.

Investigation of adult neurogenesis is complicated, as unique markers of NSCs are lacking (Chojnacki et al., 2009). Nestin and Sox2 are the most popular biomarkers for NSCs, but Nestin is particularly useful because it is expressed predominantly in brain NSCs, being absent from virtually all other adult brain cells (Dahlstrand et al., 1995; Gilyarov, 2008). Dcx, the cell phenotype of a juvenile neuron, is a microtubule-associated protein that regulates process of polymerization and cytoskeleton stabilization in neuroblasts during migration (Nkomozepi et al., 2019). In this present study, we found that CSF-cNs expressed the NSC markers Sox2 and Nestin, as well as the immature neuronal marker Dcx, indicating that these CSF-cNs may be a potential candidate of NSCs *in vivo*.

Previous studies have shown that NSCs undergo proliferation in response to mitogen stimulation (Craig et al., 1996; Martens et al., 2002; Coskun et al., 2008). Additionally, our study shows that the application of EGF and FGF-2 promotes the expansion of CSF-cNs *in vitro*. Moreover, CSF-cNs sphere-forming assays indicated that these cells could be passaged to 4th generation and proliferated in a medium containing EGF and FGF-2. The CSF-cNs expressed the NSC markers Nestin, Sox2, and Gfap. Aside from this, they could differentiate into neurons, astrocytes, and oligodendrocytes in the neuronal differentiation medium. Some studies have shown that angiogenic factors can activate NSCs (Calvo et al., 2011; Han et al., 2015; Liu et al., 2019). In line with this, following intracerebroventricular injection of VEGF and bFGF, our study also showed that the proportion of CSF-cNs expressing the NSC markers Nestin and Gfap was higher in the VEGF and bFGF group than the control group. In addition, the ratio of Pkd2l1^+^ Ki-67^+^ co-expressed cells significantly increased in the group injected with VEGF and bFGF. Finally, in this group, the proportion of Dcx^+^ Pkd2l1^+^ co-labeled cells were also markedly higher than the control group, implying that these cells have the properties of NSCs.

Accumulating evidence indicates that neural stem or progenitor cells can be activated by injury stimulation and undergo proliferation and differentiation (Jin et al., 2001; Arvidsson et al., 2002; Poplawski et al., 2020). One study suggested that the number of Nestin-positive cells reached a peak on the seventh day after SCI (Xue et al., 2021). However, our study demonstrated that the expression of Nestin reached a peak at 3 days post injury. Moreover, the expression of Pkd2l1 in the SCI groups was also higher than the sham groups. Indeed, this study showed that more Pkd2l1^+^ cells co-expressed the NSC markers Nestin and Gfap in the SCI group than the sham group. Moreover, the ratio of Pkd2l1^+^ Ki-67^+^ co-staining cells also significantly increased in the SCI group, which means that a part of the co-expressing cells underwent proliferation. Most proliferating cells were located in the ependymal cell layer. We also observed a small population of proliferating cells outside the ependymal region. In a previous study, Pkd2l1 positive cells migrated during the growth and development (Tonelli Gombalová et al., 2020). These cells may be derived from the NSCs of the ependymal cell layer, and its intrinsic mechanism needs to be explored in future research. Finally, we observed a visibly increased proportion of Dcx^+^ positive cells in the SCI group. Therefore, our results show that SCI promotes the proliferation of CSF-cNs in the spinal cord. Additionally, these findings demonstrate that CSF-cNs can be activated after SCI.

In view of the present findings, this study suggests that CSF-cNs in the spinal cord are NSCs. The present study also suggests that CSF-cNs-derived NSCs in the central canal may act as sources for supplying neuronal cells to injured regions. Therefore, further studies focused on the molecular mechanisms that regulate or perturb CSF-cNs proliferation and differentiation would be useful to develop the therapeutic potential of CSF-cNs for SCI.

## Data availability statement

The raw data supporting the conclusions of this article will be made available by the authors, without undue reservation.

## Ethics statement

The animal study was reviewed and approved by Experimental Animal Center of Guizhou Medical University.

## Author contributions

QL and X-WD designed the experiments. LC, QZ, M-ZH, Z-RL, YZ, P-JA, L-LY, WT, and C-QW performed the experiments. LC and M-ZH performed the data analysis and wrote the manuscript. All authors contributed to the article and approved the submitted version.
